# Evaluation of a Physical-Psychological Integrative (PPI) intervention for community-dwelling spinal cord injury survivors: Study protocol of a preliminary randomized controlled trial

**DOI:** 10.1371/journal.pone.0282846

**Published:** 2023-03-20

**Authors:** Yan Li, Arnold Wong, Wai Man Chung, Mengqi Li, Alex Molasiotis, Daniel Bressington, Christina Zong-Hao Ma, Patrick Pui Kin Kor, Wing Fai Yeung

**Affiliations:** 1 School of Nursing, The Hong Kong Polytechnic University, Hong Kong, China; 2 Department of Rehabilitation Sciences, The Hong Kong Polytechnic University, Hong Kong, China; 3 Virtus Medical Group, Hong Kong, China; 4 College of Arts, Humanities and Education, University of Derby, Derby, United Kingdom; 5 College of Nursing and Midwifery, Charles Darwin University, Darwin, Australia; 6 Department of Biomedical Engineering, The Hong Kong Polytechnic University, Hong Kong, China; Nnamdi Azikiwe University, NIGERIA

## Abstract

**Introduction:**

There is a considerably large group of community-dwelling spinal cord injury (SCI) survivors living with low quality of life. Physical inactivity, depression, and chronic pain are major problems faced by SCI survivors discharged from the acute phase of treatment or inpatient rehabilitation. This study aims to evaluate the feasibility, acceptability, and preliminary effects of a Physical-Psychological Integrative (PPI) online group intervention on community-dwelling SCI survivors’ physical activity, depression, and chronic pain.

**Methods:**

This is a two-arm pilot randomized controlled trial with repeated measures (pre-, post-intervention, and 3-month follow-up) design. Seventy-two participants will be randomly assigned to two study groups. The PPI intervention group will receive a video program for physical activity training and eight-week online group psychological interventions using skills of group-based motivational interviewing and mindfulness-based stress reduction. The control group will receive an eight-week online didactic education programed. Focus-group interviews will be conducted post-intervention to explore their views about acceptance and suggested improvements to the intervention. The feasibility of study procedures and the acceptability of interventions will be evaluated. The effectiveness of the PPI intervention will be evaluated by leisure-time physical activity, depression, chronic pain, exercise efficacy, mindfulness, and quality of life. We will use the generalized estimating equation to assess intervention effects and content analysis for interview data. This study has received ethical approval from the Hong Kong Polytechnic University (HSEARS20210705004) and was registered in ClinicalTrials.gov (NCT05535400).

**Discussion:**

This study will be the first to provide empirical data on the evaluation of an online-group intervention integrating both physical activity promotion and psychological approaches, aimed at reducing physical inactivity, depression, and chronic pain for community-dwelling SCI survivors in Hong Kong. The findings could provide evidence supporting the use of PPI intervention as a novel online group support, in addressing both the physical and psychological needs of community-dwelling SCI survivors.

## Introduction

Spinal cord injury (SCI) is a neurological disorder that leads to partial or complete loss of people’s motor and/or sensory function below the level of the injury [1]. According to the latest data from the Global Burden of Disease Study, there were estimated to be 0.9 million incident cases, 20.6 million prevalent cases, and 6.2 million years lived with disability of total SCI in 2019 globally [2]. The age-standardized prevalence rate of SCI presented an increasing trend with 0.1 estimated annual percentage change from 1990 to 2019 [2]. In Hong Kong, there is no central registration for the exact number of SCI survivors. It is estimated there are currently thousands of SCI survivors living in communities and around 200 new cases of traumatic SCI every year in Hong Kong [3]. Most new SCI cases result from car accidents, industrial accidents, or falls [4]. Older adults are also prone to SCI as their degenerated spines are vulnerable to severe injuries [5]. SCI imposes significant physical and psychosocial burdens on survivors, their relatives, and the medico-social system as a whole. Importantly, the economic burden is substantial due to the permanent disability, various SCI-related chronic conditions, and injury occurrence at a younger age [6]. The direct cost of care including rehabilitation for the first year after SCI ranged from 32,240 to 1,156,400 US dollars, and for ensuing years from 4,490 to 251,450 US dollars in a systematic review of 30 studies from 8 countries or regions [7].

Currently, there is no effective treatment to cure SCI [8]. Survivors often suffer immediate and irrecoverable neurological loss and severe disabilities that last the rest of their lives [1]. Individuals with SCI experience various secondary physical and psychosocial consequences during their long recovery process [9]. Physical inactivity, depression, and chronic pain are the major challenging problems placing detrimental effects on the quality of life and well-being of people with SCI [10]. Approximately 50% of SCI survivors have no engagement in leisure-time physical activities after discharge from inpatient rehabilitation, and this inactivity is linked with increased risks for depression, pain, and poor quality of life [11, 12]. Individuals’ physical activity could be determined by perceived self-efficacy over the control of exercise [13]. Those with lower exercise self-efficacy indicated lower engagement in physical activities [14]. Depression is the most common mental health problem post-SCI, with a prevalence rate ranging from 7.0% to 47.7% [15]. Depression interferes with the ability of individuals to perform daily activities and affects their quality of life [16, 17]. Chronic pain (persisting for more than 12 weeks) also remains a significant problem during the long-term rehabilitation of people with SCI, with an estimated prevalence rate of 68% [18]. Chronic pain imposes significant negative impacts over quality of life, physical and emotional functioning of people with SCI [12, 19].

A systematic review of 12 clinical trials of activity-based interventions for people with SCI has demonstrated positive effects on people’s mobility, independence, and quality of life [12]. However, only half of these studies were randomized controlled trials (RCTs), and most were conducted in the inpatient rehabilitation or the sub-acute phase of post-SCI people. Limited high-quality trials have focused on the long-term rehabilitation outcomes (e.g., physically active lifestyle, chronic pain, and psychological well-being) of people with SCI living in the community [20, 21]. One reason for the lack of trials might be due to barriers of transportation and lack of face-to-face interaction with healthcare professionals [22]. Two pilot trials that integrated community-based physical activities and telephone counseling from physiotherapists indicated positive effects of an activity-based intervention on functional improvement and physical fitness for community dwellers with SCI [23, 24]. In addition, previous research also indicated positive effects of physical activities on SCI survivors’ mood (e.g., depression) and subjective well-being [12]. People’s adherence to the practice of physical activities after community reintegration remains a big challenge and studies have highlighted the importance of psychological interventions (i.e., motivational interviewing) in enhancing people’s motivation and adherence to physical activity [23, 25]. Telephone counseling and online face-to-face meetings may be good modalities for overcoming the barriers of not having face-to-face interactions and transportation problems.

Psychological approaches, as a typical non-pharmacological intervention for treating depression and pain post-SCI, are effective alternatives/adjunct therapies to pharmacological interventions which are accompanied by various side effects [26, 27]. Psychological interventions (e.g., cognitive behavioural therapies, mindfulness interventions) have been given much attention in recent research and have shown positive effects in relieving depression, pain intensity, and pain-related disability of people with SCI [28–30]. In addition, psychological interventions can enhance people’s self-efficacy and confidence in facing various challenges and stressful situations post-SCI, as well as improve engagement in physical exercise and rehabilitation [31]. Prior research has shown two main waves of psychological interventions for the SCI population, including cognitive behavioural therapies and coping skills-based strategies [32]. The most recent focus (the third wave) is to apply acceptance- and mindfulness-based interventions, in order to cultivate ‘acceptance’ to thoughts, behaviours, and emotions [33]. Mindfulness, a central construct of third-wave therapies, is defined as ‘awareness that arises through paying attention, on purpose, in the present moment, non-judgmentally [33]. A recent systematic review of five mindfulness interventions for people with SCI indicated a significant reduction in depressive symptoms and pain-related outcomes [30]. However, conclusions are preliminary given the small number of included studies and moderate-to-high risk of bias in these studies (e.g., small sample sizes or lack of a control group). That said, the current evidence highlights the potential for mindfulness interventions in improving outcomes of depression and chronic pain, and the need for more high-quality studies in this area.

In summary, there is a considerably large group of community-dwelling SCI survivors in Hong Kong who have low quality of life [34]. Physical inactivity, depression, and chronic pain are major problems faced by SCI survivors discharged from the acute phase of treatment or inpatient rehabilitation [10]. Despite the potential benefits of psychological strategies to increase the efficacy of activity-based interventions, there is a scarcity of studies to explore the possible effects that interventions incorporating both physical and psychological components may have on community-dwelling SCI survivors. Particularly, none is reported in Hong Kong. Further, people with SCI living in the community may have great difficulties in engaging in face-to-face group intervention due to their reduced physical function. Given the above, our study will be the first to develop an online-group intervention that includes both physical activity promotion and psychological approaches (i.e., mindfulness-based skills training and SCI-related psychoeducation) to address the health needs of community-dwelling SCI survivors living in Hong Kong. Specifically, the physical activity component will address participants’ rehabilitation needs by enhancing their physical activity and body functions. In contrast, the psychological component will promote their subjective well-being and community participation. The integration of the physical and psychological approaches can truly fulfill community-dwelling SCI survivors’ needs, as well as fill the research gap of lacking high-quality RCTs. Our findings will lay the foundations for a fully-powered RCT which would be a reference for policymakers to develop innovative and comprehensive community healthcare for people with SCI.

## Methods

### Aims and research hypotheses

This study aims to evaluate the feasibility, acceptability, and preliminary effects of an eight-week Physical-Psychological Integrative (PPI) online-group intervention on physical inactivity, depression, and chronic pain for people with SCI living in the community. We hypothesize when compared with those in the control group (online telephone didactic education), participants in the PPI intervention group will demonstrate significantly greater improvements at post-intervention and 3-month follow-up in (1) minutes of performing the moderate-to-rigorous physical activity as well as the number of days they are physically active for a total of at least 60 minutes or more per day; (2) depression and chronic pain; and (3) mindfulness skills and quality of life.

### Design

**Fig 1:**
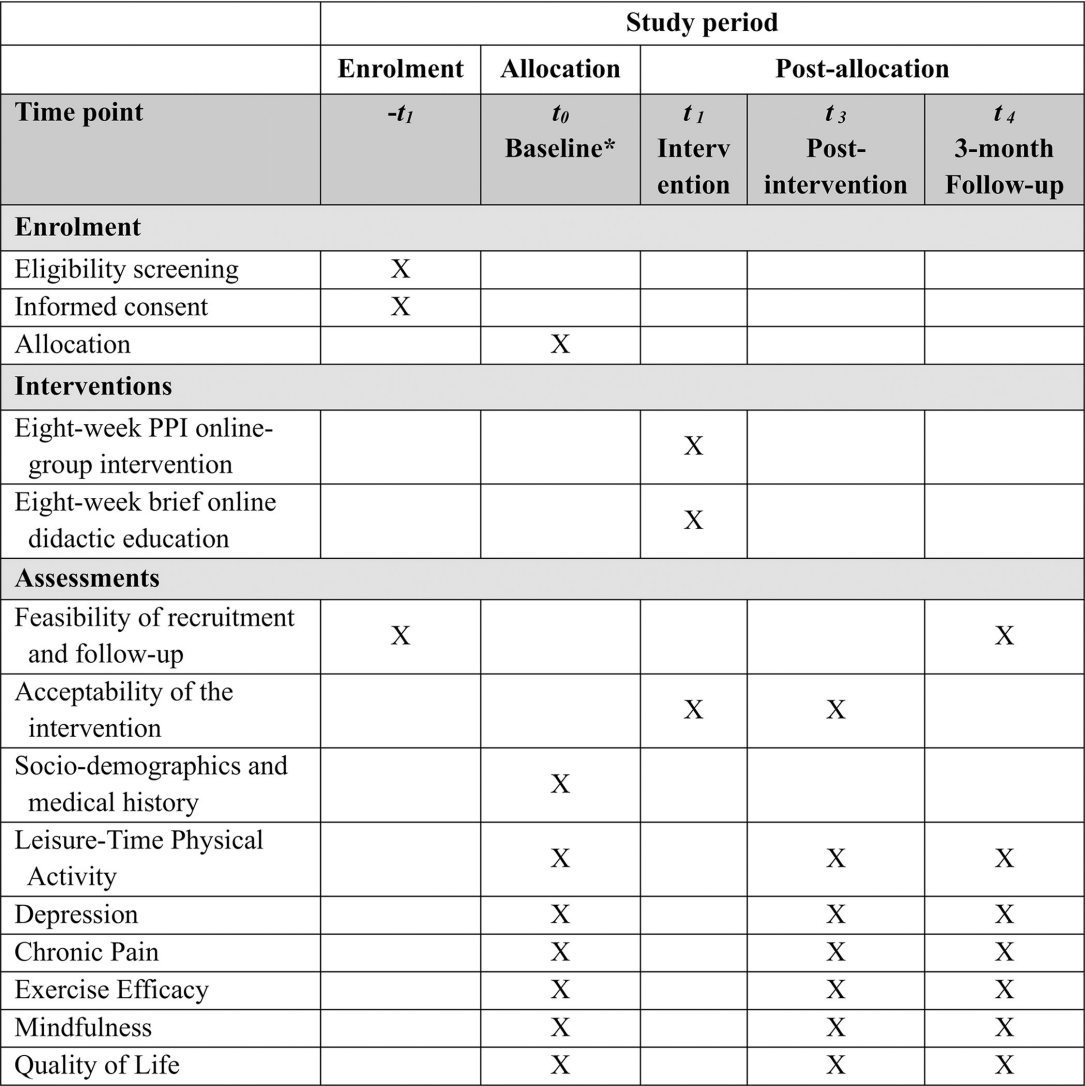
SPIRIT schedule of enrollment, interventions, and assessments.

**Fig 2:**
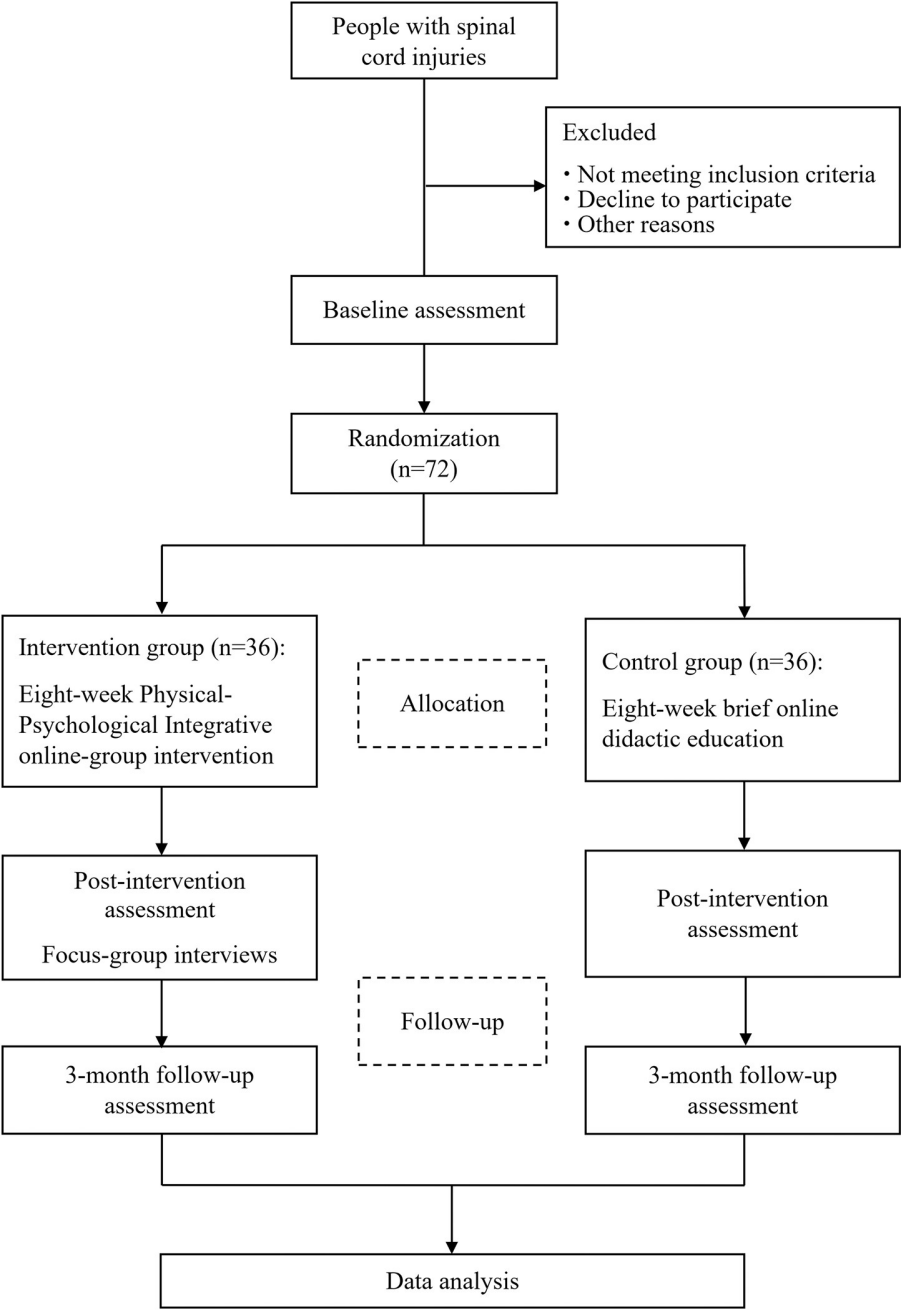
Flow chart of the study.

An open-label, two-arm pilot RCT will be conducted to investigate the effects between the intervention (PPI online-group intervention) and the control group (brief online didactic education) for people with SCI living in the community over a 3-month follow-up. Qualitative interviews with focus groups involving participants in the PPI intervention group will be conducted post-intervention, to explore participants’ views about acceptance, strengths and limitations, and suggested improvements of the intervention. This study has been registered in ClinicalTrials.gov (NCT05535400). The trial was designed in conformance with the Standard Protocol Items: Recommendations for Interventional Trials (SPIRIT). A SPIRIT schedule of enrollment, interventions, and assessments is shown in Fig 1. The flow chart of the study is presented in Fig 2.

### Participants and sample size

#### Participants

Study participants will be recruited from the Hong Kong Direction Association for the Handicapped and other community centers in Hong Kong, where groups of people with physical impairments can be reached. Emails/phone call invitations will be sent to potential participants and advertising posters will also be used to encourage them to participate.

People with SCI will be included if they are: (1) at least 18 years old; (2) currently living in the community and having SCI for more than 6 months; (3) complete injury at the C6 or below or incomplete injury at any level; (4) having a computer/smartphone with audio-speaking function and Zoom software, and internet access in a secure place; (5) using a wheelchair for at least 2 hours a day and having approval from their physicians to perform exercises; and (7) able to communicate in Cantonese and to provide informed consent.

People with SCI will be excluded if they are: (1) presenting with any significant cognitive impairment or brain injury; (2) having problems in hearing, verbal communication and vision; (3) engaged in ongoing psychotherapy or any other physiotherapy/exercise/relaxation interventions; (4) physically active for more than 150 minutes per week; and (5) experiencing significant psychotic symptoms, substance misuse or medically unfit for the exercise and psychological programme as diagnosed by their physicians.

#### Sample size

The central limit theorem indicates a minimum sample of 30–40 that is sufficient to estimate the mean (M), standard deviations (SD), and 95% Confidence Intervals [35]. As this proposed study is to develop and evaluate the feasibility, acceptability, and preliminary effectiveness of the PPI intervention, a widely acceptable sample size for a preliminary analysis will be adopted to examine intervention feasibility and to estimate a between-group effect (i.e., 30 participants in each study group for a small standardized effect size of 0.25 with 80% power and two-sided 5% significance) [36]. Considering a potential 20% attrition rate, 36 people with SCI per study group (i.e., 72 people in total) will be recruited. A purposive sampling by key informants (containing both participants who completed all the intervention sessions and those who withdrew from the intervention) will be conducted for focus-group interviews (i.e., 4–5 participants per group), until saturation of the information was reached as no relevant new codes will be found in data [37].

### Procedures and data collection

A research assistant, trained by a senior researcher from the research team, will be responsible for recruitment and data assessment at baseline, post-intervention, and 3-month follow-up. At the time of enrollment, eligibility screening and obtaining informed consent will be achieved. The baseline assessment will be performed after recruitment and before random allocation. This initial assessment will be conducted face-to-face or online if participants cannot come to the study site. Questionnaires for assessment will be self-completed by participants. Assistance for those who have difficulties in filling out surveys will be provided by their caregivers or the research assistant. The baseline assessment will include participants’ socio-demographics, medical history, and clinical outcomes.

After the completion of the baseline assessment, eligible participants will be randomly allocated to two study groups. The random allocation will be conducted by an external randomization service (i.e., sealedenvelope.com) to ensure allocation concealment and will use random permuted block sizes (i.e., 4 or 6) to ensure balanced group numbers. Once a participant consents to take part and completes the baseline assessments an administrative clerk outside the research team will enter their unique participant number into sealedenvelope.com and the participant’s group allocation will be revealed. The clerk will then inform the research team which group the participant is allocated to. Participants will not be blinded due to the nature of the intervention. Participants will be reminded to keep the intervention content confidential and not discuss this with participants in the control group to avoid contamination. The research assistant will contact participants via phone/WhatsApp and encourage them to attend the online meetings as scheduled. Fitbit wristband activity trackers will be used as incentives for the participants to remain in the study. Intervention sessions will be audio-taped with participants’ consent for reviews and discussion of improvements among the research team. Further assessment of clinical outcomes will be conducted online at post-intervention. Focus-group interviews at post-intervention will be carried out online to assess the acceptability of the PPI intervention.

The online final assessment will be conducted three months after intervention as the follow-up assessment. The investigator will use the same set of outcome questionnaires at pre-, post-intervention, and 3-month follow-up. The feasibility of recruitment and follow-up will be assessed at enrollment and 3-month follow-up. The acceptability of the intervention will be assessed during and at post-intervention.

### Assessments

#### Socio-demographics and medical history

A self-designed form will be used to collect data on participants’ socio-demographics and medical history, including age, gender, employment, marital status, ethnicity, cause, level of injury, and time since the injury.

#### Feasibility and acceptability

The feasibility assessment of subject recruitment and follow-up will include (1) time taken to recruit the target sample size (time period for recruitment will be around 6 months based on existing literature to recruit people with SCI) [38–40]; (2) recruitment rate: the number of eligible participants approached who agree to consent divided by all subjects eligible to participate; (3) retention rate: the number of participants who complete the study divided by all participants who agree to consent; (4) drop-out rate: the number of participants who dropped out after randomization divided by all participants who agree to consent; and (5) reasons for dropping out will be collected from participants who dropped out.

The acceptability of the intervention will be indicated by (1) adherence rate: the number of PPI intervention sessions practiced divided by the total number of required sessions; (2) adverse events record associated with PPI intervention; and (3) satisfaction: participants’ satisfaction with the intervention will be assessed with a general scale, the 8-item Client Satisfaction Questionnaire with grading on a four-point Likert scale [41]. The scores will be summarized with higher scores indicating greater satisfaction. The questionnaire has shown good reliability (Cronbach’s *α* = 0.94) in the SCI population [42]. The acceptability of the intervention will also be assessed by focus-group interviews with a purposive sample of participants who completed all the intervention sessions and those who withdrew from the intervention. Participants’ perspectives about the acceptability, strengths, and limitations of the study and suggestions on the improvements of the interventions will be explored.

#### Effectiveness of the PPI intervention

**Fig 3:**
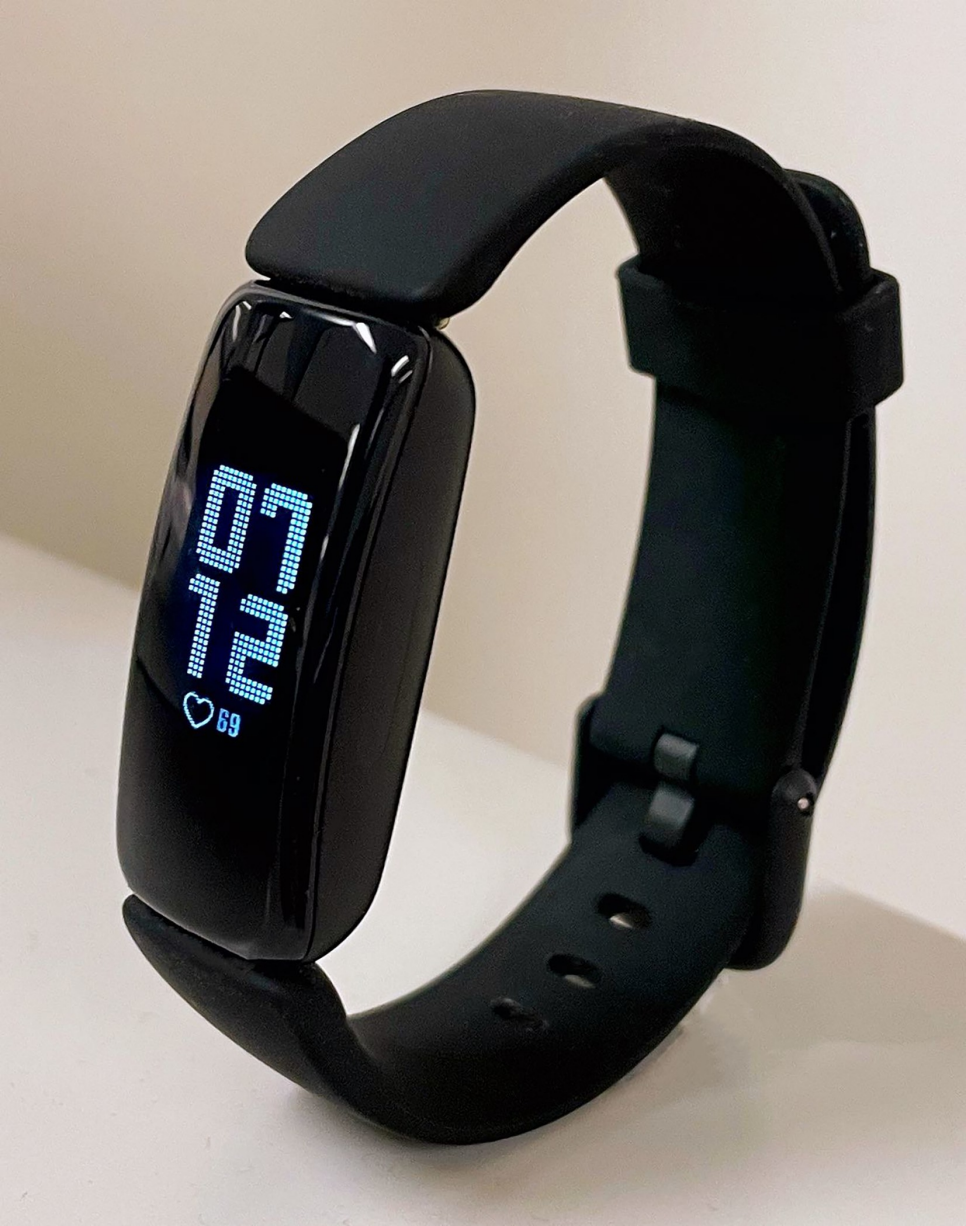
Fitbit Inspire 2^®^ (San Francisco, IL).

*Primary outcomes*. Leisure-Time Physical Activity. Participants’ leisure-time moderate-to-rigorous physical activity will be assessed by using the Fitbit Inspire 2^**®**^ (San Francisco, IL) which is a wearable, display, triaxial accelerometer that pairs with Bluetooth with compatible smartphones via Bluetooth (Fig 3). It can monitor the heartbeat rate of users for each minute. The heart rate zone for moderate-to-rigorous physical activity ranges from 64% to 93% of the maximum heart rate [43], which is often restricted to 130 beats per minute in individuals with tetraplegia [44]. Participants will be asked to wear the Fitbit tracker for more than 10 hours a day (defined as daily adherence) throughout the study period. This accelerometer-based wristband activity tracker has shown strong criterion validity (rho/kappa≥0.90) in measuring adults with wheelchairs and walking aids [45].

Depression. The 9-item Patient Health Questionnaire, one of the most commonly used self-administered depression screening tools, will be used to measure the participants’ depression level [46]. Participants will be asked to rate “how often they were bothered by specific symptoms over the last two weeks” with each item rating from 0 (not at all) to 3 (nearly every day). Higher scores indicate greater symptoms of depression. The scale has indicated satisfactory reliability in the British SCI population [26] (Cronbach’s *α* = 0.92) and the Chinese general population (Cronbach’s *α* = 0.86) [47].

Chronic pain. Pain intensity will be assessed using the Numerical Pain Rating Scale. This tool is an 11-point numerical pain rating scale, where zero indicates no pain and 10 indicates the worst imaginable pain (in the past week) [48]. The scale has demonstrated good test-retest reliability (intraclass correlation coefficient = 0.99) in the Chinese SCI population [49].

*Secondary outcomes*. Exercise efficacy. The 9-item Chinese version of the self-efficacy for exercise will be used to evaluate participants’ confidence level (from 0 not confident to 10 very confident) regarding engaging in regular exercise [50]. The average scores of ten items will be calculated, with the higher scores indicating greater exercise efficacy. The scale demonstrated acceptable reliability (Cronbach’s *α* = 0.75) in Chinese adults [50] and it was tested in Chinese people using wheelchairs [51].

Mindfulness. The 39-item Five Facet Mindfulness Questionnaire, a comprehensive and commonly used tool for mindfulness evaluation, will be used to measure the five aspects of mindfulness including observing, describing, acting with awareness, non-judgmental and non-reactive [52]. Answering on a five-point Likert scale (from 1 never to 5 always true), higher scores denote greater levels of mindfulness. Satisfactory reliability was found for each subscale in the British SCI population (Cronbach’s *α* ranged from 0.89 to 0.92) [26] as well as the Chinese community sample (Cronbach’s *α* ranged from 0.66 to 0.91) [53].

Quality of Life. The 26-item World Health Organization Quality of Life Brief Scale is a widely used tool for assessing participants’ quality of life in four domains including physical health, psychological health, social relationships, and environment [54]. Higher scores indicate greater perceived quality of life with grading on a five-point Likert scale. This scale has demonstrated strong psychometric characteristics of good reliability in the British SCI population (Cronbach’s *α* = 0.96) [26] and acceptable content validity in the Chinese context (r = 0.39–0.65) [55].

### Interventions

The Intervention Group: The eight-week Physical-Psychological Integrative online-group intervention. The PPI intervention consists of two major parts, i.e., the first part of the physical exercise and the second part of the 8-week online intervention. For the first part of the PPI intervention, a guiding video of performing exercises was specifically developed for the SCI population by two experienced physiotherapists in Hong Kong. The video displays 25-minute aerobic exercises, mainly including warm-up, mobilization, strength exercises, aerobic exercises, and cool-down sessions. Participants will be encouraged to follow the video to practice exercises on a daily basis. The practice lasts for 8 weeks in total.

The second part of the PPI intervention includes eight weekly online-group sessions (with 4–5 participants in a group and each session lasting for 60–90 minutes). At the beginning of each online group meeting, the intervention provider will use motivational interviewing techniques [56] to promote participants’ adherence to the physical activity program mentioned in Part I, followed by online group psychological intervention. The content of the intervention will be adapted from the mindfulness-based stress reduction proposed by Kabat Zinn’s (1992), practice guideline ‘mindfulness for health’ to relieve pain, reduce stress, and restore well-being [57]. The content of the intervention includes eight themes/sessions (the details of the intervention content are shown in S1 Appendix): Session 1- Orientation and engagement; Session 2- Awareness and Acceptance; Session 3- Non-judgement; Session 4- Stay present and let go; Session 5- Our thoughts are not real & response without reacting; Session 6- Empowerment of self-management and discuss pain management; Session 7- Seek out pleasant things and social support; Session 8- Review the intervention and end the programme. Audio-recorded sessions (i.e., 4–5 minutes each) of mindfulness practice (mindfulness meditation and mindfulness exercises) will be given to the participants for their daily practice after they have completed each of the real-time online session. The intervention will be carried out by an experienced social worker trained in psychology, motivational interviewing, and mindfulness-based stress reduction.

Participants in the control group will receive a video call (approximately 60 minutes each week for eight weeks) from the trained research assistant with a psychological background, i.e., to provide general physical and psychological suggestions (e.g., encouragement to perform physical activities, and communication skills with family members/friends, and engagement in community life). This is to control the contact effects of the PPI intervention.

### Data management

A detailed database will be set up to record each participant’s assessments and progress. A unique subject identifier will be assigned to each participant for confidentiality. The database will be password-protected and will only be accessible to the research team. Ten percent of cases will be randomly audited for data entry review and quality checks. A researcher independent of the study execution will be responsible for periodic monitoring of data collection progress, quality, and safety. All adverse events will be identified, reported, and handled cautiously. The discrepancy will be discussed with our research team (physiotherapist, doctor, rehabilitation nurses, and academic staff) first to reach a group consensus, and consultation with experts in clinical trials will be conducted if necessary.

### Data analysis

All quantitative data from the pre- and post-tests will be numerically coded, summarized (with descriptive statistics), and analyzed using IBM SPSS 26. Both intention-to-treat (to evaluate the effect of assigning a treatment) and per-protocol analyses (to evaluate the effect of receiving a treatment) will be performed and compared [58]. Effect sizes of between-group comparisons will be estimated using Cohen’s *d* which is applicable for small sample sizes [59]. Intervention effects will be analyzed using the generalized estimating equation model, which can account for data missing at random [60]. Group, time, and their interaction effects could be analyzed using this model. Randomized testing between the intervention and control group at baseline will be conducted to identify potential covariates. Specifically, the Chi-squared test will be used for comparisons of categorical variables. Two samples independent *t*-test and Mann–Whitney *U* test will be conducted with continuous variables for normal and skewed distributions, respectively. Co-variance (if any found in the baseline comparison) will be adjusted during the analysis of intervention effects. Subgroup analysis will be conducted for certain factors (e.g., complete and incomplete injury or intervention completion and non-completion). The level of significance is set at *p*<0.05 (2-tailed).

Qualitative data generated from focus-group interviews will be content analyzed. Tape-recorded interviews will be transcribed into Cantonese by one research assistant and cross-checked by the other research assistant. The transcripts will then be independently coded by a research assistant with training in qualitative content analysis, and important manifest contents and latent meanings in the data will be identified. Codes will be discussed and agreed between our team members, combined to form categories/subcategories, and explained with verbatim data. Any discrepancy in coding/category will be discussed and final themes/subthemes will be confirmed by our team.

### Ethics considerations

This study has been approved by the Human Subjects Ethics Committee at the Hong Kong Polytechnic University (HSEARS20210705004) and will be performed in accordance with the Declaration of Helsinki. Written informed consent will be obtained from all participants before the baseline assessments of, and will also be obtained from purposely sampled participants prior to all focus-group interviews. Participation is entirely voluntary for people with SCI and they can withdraw from the research at any time without any penalty. Participants’ identities and data (information collected and videotape records) will be protected by maintaining anonymity and confidentiality. Only authorized personnel can access the data for analysis.

## Discussion

Our study will be the first RCT to test the feasibility, acceptability, and preliminary effectiveness of a psychological and physical integrated intervention for community-dwelling spinal cord injury patients in Hong Kong. This study addresses several important health issues that are currently immature for people with SCI living in the community.

In particular, our PPI programme incorporates psychological skills (i.e., motivational interviewing techniques), which can improve participants’ adherence level to physical exercises [23]. Also, further research evidence will consolidate the potential positive effects of Mindfulness interventions, which is the third wave of psychological interventions that have shown the potential to reduce depression and chronic pain for people with SCI [30]. Our findings can address the community-dwelling SCI population’s health needs and have the potential for improving community healthcare services for this client group. Importantly, our findings will directly inform the future development and implementation of a large-scale clinical trial in producing robust research evidence in exploring the effects of PPI programmes for the SCI population.

This study also has some limitations. Due to the nature of the intervention, participants cannot be blinded to interventions. Assessor-blinding is not adopted as measurements are self-reported. Assistance provided by caregivers and research assistants might be needed for those who have physical difficulties in filling out surveys. The relatively small sample size limits the internal and external validity of the study findings. The medium length of follow-up does not allow us to evaluate the long-term ongoing effects of the PPI programme. Nonetheless, our preliminary RCT can inform future high-quality full-scale clinical trials and address the limitations well.

## Dissemination

The study results will be published in peer-reviewed scientific journals. Participants at enrolment will be asked if they would like to receive any results or publications from this study. The findings will also be disseminated at major national and international academic conferences or seminars. A briefing report will be distributed to organizations to share knowledge and guide healthcare programmes for the broader SCI population. The potential organizations will include the Department of Health of the Hong Kong Special Administrative Region and non-government organizations (e.g., the Hong Kong Direction Association for the Handicapped).

## Supporting information

S1 Checklist SPIRIT checklist. (DOCX)

S1 Appendix Details of the intervention. (DOCX)

S1 Protocol Project submitted to the ethics committee. (PDF)
