# Supplementary material for: Evaluation of a Physical-Psychological Integrative (PPI) intervention for community-dwelling spinal cord injury survivors: Study protocol of a preliminary randomized controlled trial
Source: PLoS One. 2023 Mar 20;18(3):e0282846. doi: 10.1371/journal.pone.0282846 (PMC10027219; doi:10.1371/journal.pone.0282846)
Supplement: S1 Appendix — (DOCX) [file pone.0282846.s002.docx]

| **S2 Appendix. Details of the intervention.** | | | |
| --- | --- | --- | --- |
| **Session** | **The theme of the session** | **Objectives** | **Content** |
| **1st session** | Orientation and engagement | - Get familiar with the use of the online platform - Understand the aim/objectives of the intervention - Give practical information about group meetings - Encourage participants’ attendance at the programme - Review and discuss the physical activity practice | - Introduction of the online platform, facilitate and group members. - Explain the aim of the intervention to increase their activity levels, and reduce depression and chronic pain - To state the group rules and meeting details (attendance, respecting others and being quiet when practising) - Homework: physical activity practice |
| **2nd session** | Awareness and Acceptance | - Discuss the practice of physical activities - Practice body scans draw notice to the body sensations, rather than attending to thoughts, ideas or fears about the sensations - Discuss disease management in the community | - Body scans of guiding the participants to draw their attention to various areas of the body; move awareness systematically through each area of the body, and notice sensations of the body in a precise and detailed manner, as opposed to attending to thoughts, ideas or fears about this sensation. - To discuss disease management in the community, and the importance of psychosocial care - Homework: physical activity, body scan, and mindful breathing meditation |
| **3rd session** | Non-judgement | - Discuss the practice of physical activities - Explore present moment experience - Focused attention on breathing with acceptance and a non-judgmental attitude - Practice breath awareness meditation | - Breath awareness meditations began with a broad awareness of the bodily experience of breathing, becoming increasingly focused on more subtle aspects of breathing and encouraged participants to notice when their attention wandered away from the meditation - Homework physical activity, body scan and mindful breathing |
| **4th session** | Stay present and let go | - Discuss the practice of physical activities - Practice the attitude: Stay present and let go - Increase acceptance and compliance with medication - Practice mindfulness movements and mindful sitting | - Mindfulness movement and mindful sitting (with breath awareness) - Observe experience with stay present attitude and observe body sensations, feelings and thoughts - Homework: mindful movement and mindful sitting, physical activity |
| **5th session** | Our thoughts are not real & Response without reacting | - Discuss the practice of physical activities - Aware of the thoughts—alternative perspectives of seeing your thoughts and sensations - Integrate mindfulness in managing stress and daily difficulties - Recognize recurring thoughts and stand back from them, without questioning them - Discuss stigma and how to react to discrimination | - Continue the practice of body scans and mindful breathing - Recognize negative thoughts - An alternative response to negative thoughts is “mindful, and response without reacting” - Discuss stigma and how to react to negative thoughts and behaviours to discrimination - Homework: recognize negative thoughts and practice mindfulness response without reacting |
| **6th session** | Empowerment of self-control | - Discuss the practice of physical activities - Use a mindfulness attitude for problem-solving and communication with mindfulness attitude | - Discuss participants’ problems, and how to perform problem-solving - Learn to use a mindfulness attitude for communication - Homework: practice problem-solving skills and mindfulness communication |
| **7th session** | Seek out pleasant things | - Encourage the exploration of pleasant things in life - Practice kind mediation | - Seek out the pleasant things in life that pain and other sufferings may have prevented from appreciating, aa and find a particular time in a day to find/do things positively. - Meditation that encourages kindness to themselves and others and relaxes into the pain instead of being distressed by it |
| **8th session** | Review the programme and end the session | - To encourage participants to persist in practising leisure time activities, and to practice, skills learnt in the mind-body intervention - To build social networks and share the experience with peers - To end the programme | - Invitation to outcome assessment - Importance of social networking and sharing experience with peers - Review the mindfulness skills and encourage the continuous practice of physical activity and mind-body interventions - To end the session |
