# Supplementary material for: Evaluation of a Physical-Psychological Integrative (PPI) intervention for community-dwelling spinal cord injury survivors: Study protocol of a preliminary randomized controlled trial
Source: PLoS One. 2023 Mar 20;18(3):e0282846. doi: 10.1371/journal.pone.0282846 (PMC10027219; doi:10.1371/journal.pone.0282846)
Supplement: S1 Protocol — (PDF) [file pone.0282846.s003.pdf]

Protocol Title	Effects of a Physical-Psychological Integrative (PPI) Intervention on Physical Inactivity, Depression and Chronic Pain for Community-Dwelling Spinal Cord Injury Survivors: A Pilot Randomized Controlled Trial
**Principal Investigator**	Yan Li

a) Introduction

Spinal cord injury (SCI) is a neurological disorder that leads to "partial or complete loss of people's motor and/or sensory function below the level of the injury" [1]. A review of the epidemiology of worldwide SCI reported that the incidence lied between 13 and 163.4 per million people, and the global prevalence ranged from 490 to 526 per million among developed counties [2]. The "International Campaign for Cures of Spinal Cord Injury Paralysis" stated that a conservative estimation of the worldwide prevalence of people with SCI-caused paralysis is over 2.5 million [2]. In Hong Kong, there is no central registration for the exact number of SCI survivors. According to the information of Hospital Authority (https://www.ha.org.hk/) and non-government organization (https://www.4limb.org/), there are currently thousands of SCI survivors living in the community, and the total SCI population is increasing with around 200 new cases of traumatic spinal cord injuries every year in Hong Kong. Most of the new SCI cases result from car accidents, industrial accidents, or falls. Older adults are also prone to SCI as their degenerated spines are vulnerable to severe injury. Importantly, various SCI-related chronic conditions impose a huge economic burden (costs ranging from US$ 68,000 to 180,000 per person per year) on the healthcare system [3].

Currently, there is no effective treatment to cure SCI, although the life expectancy of SCI survivors is now equivalent to the general population given the improvement in medical treatment. Patients often suffer immediate and

irrecoverable neurological loss and severe disabilities that last for the rest of their lives. Prior research has indicated that people with paraplegia/tetraplegia following traumatic SCI have a lower quality of life than the general Hong Kong population and they need more medical care, psychosocial support, and environment adaptation [4]. People with SCI experience various secondary physical and psychological consequences during the long recovery process. Physical inactivity, depression, and chronic pain are the major challenging problems and have detrimental effects on these people's quality of life and well-being. Approximately 50% of the SCI population have no engagement in leisure-time physical activities such as wheeling, therapeutic exercise, or equipment-assisted walking after they are discharged from inpatient rehabilitation, and this inactivity is linked with increased risk for depression, pain, and poor quality of life [5]. Physical inactivity is not only determined by people's levels of injury and severity at post-SCI but also related to their behavioural patterns and psychological conditions [6]. Those with more negative emotions and low levels of self-efficacy will have lower motivations and engagement in physical activities. Depression (caused by a variety of medical, psychological, and social factors) is the most common mood problem in people with SCI, and it interferes with the ability of individuals to engage in daily life activities and affects their life satisfaction [7]. The prevalence rate of depression at post-SCI ranges from 9.8% to 63.9% at post-SCI, which is significantly higher than those of non-SCI counterparts (5.3%-11.3%) [8]. Chronic pain (persisting for more than 12 weeks) also remains a significant problem at post-SCI, with an estimated prevalence rate of around 70%-80% during long-term rehabilitation [9]. Chronic pain has a significant negative impact on the physical and emotional functioning of people with SCI, and a strong interrelationship has been found between pain and depression among people with SCI [10].

A systematic review of 19 clinical trials of activity-based interventions for people with SCI has demonstrated positive effects on people's mobility, independence, and quality of life [6]. However, most of these trials were conducted in the inpatient rehabilitation or the sub-acute phase of post-SCI. Very few trials focused on the long-term rehabilitation outcomes of people with SCI living in the community, which might be due to the barriers of transportation and lack of face-to-face interaction with healthcare professionals. Two pilot trials that integrated physical activities (e.g., home-based aerobic exercise) and telephone counselling from physiotherapists indicated positive effects of an activity-based intervention on patients' functional improvement and physical fitness [11, 12]. In addition, previous research also indicated positive effects of physical activities on SCI survivors' mood (e.g., depression) and subjective well-being [12]. People's adherence to the practice of physical activities after community reintegration remains a big challenge and studies highlighted the importance of psychological intervention (i.e., motivational interviewing) in enhancing patients' motivation and adherence to physical activity [12]. Telephone counselling and online face-to-face meetings will be good modalities for overcoming the barriers of not having face-to-face interactions and problems of transportation.

Non-pharmacological interventions (e.g., psychological approaches) for the treatment of depression and pain at post-SCI are effective alternatives/adjunct therapies to pharmacological intervention which are associated with various side effects including constipation, toxicity, and/or increased addiction/abuse [13, 14]. Psychological interventions (e.g., cognitive behaviour therapies, mindfulness interventions) have been given much attention in recent research and have shown positive effects in relieving depression, pain intensity, and

pain-related disability of people with SCI [15, 16]. In addition, psychological interventions can enhance people's self-efficacy and confidence in facing various challenges and stressful situations at post-SCI, as well as improve engagement in physical exercise and rehabilitation [15]. Prior research showed two main waves of psychological interventions for people with SCI including cognitive behaviour therapies and coping skills-based interventions/training. The most recent focus (the third wave) is to apply acceptance- and mindfulness-based interventions, in order to cultivate 'acceptance' of thoughts, behaviours, and emotions [17]. A recent systematic review of five mindfulness interventions for people with SCI indicated a significant reduction in depressive symptoms and pain-related outcomes [17]. However, conclusions are preliminary given the small number of included studies and moderate-to-high risk of bias in these studies (e.g., small sample sizes or lack of a control group). That said, the current evidence highlights the potential for mindfulness interventions in improving outcomes (i.e., depression and chronic pain), and the need of more high-quality studies in this area.

In summary, there is a considerably large group of community-dwelling SCI survivors in Hong Kong who are having low quality of life [4]. Physical inactivity, depression, and chronic pain are major problems faced by SCI survivors discharged from the acute phase of treatment or inpatient rehabilitation. Psychological factors play pivotal roles in determining people's engagement in post-discharge physical activity, as well as the management of depression and chronic pain. There is a scarcity of research that has addressed both the physical and psychological needs of community-dwelling SCI survivors in Hong Kong. Further, people with SCI living in the community may have great difficulties in engaging in face-to-face group intervention due to their reduced physical function. Online interventions (e.g., video meetings by Zoom)

may be a good alternative for community-dwelling SCI survivors to overcome the physical barriers (i.e., transportation), and to enhance their adherence to interventions. Given the above, our team will be the first to develop an online-group intervention that includes both physical activity promotion and psychological approaches (i.e., mindfulness-based skills training and SCI-related psychoeducation) to address the health needs of community-dwelling SCI survivors in Hong Kong. Specifically, the physical activity component will address participants' rehabilitation needs by enhancing their physical activity and body functions, while the psychological component will promote their subjective well-being and community participation. The integration of the physical and psychological approaches can truly meet community-dwelling SCI survivors' needs [18], as well as fill the research gaps of lacking high-quality randomised controlled trials. Previously, our team has conducted group-based psychosocial interventions (i.e., coping-oriented supportive programme) for people with SCI during inpatient rehabilitation, and the programme resulted in significant improvement of participants' coping ability, mood, and life satisfaction [19, 20].

Taken together, the proposed intervention not only can potentially promote physical activities but lower the risk of depression and chronic pain in people with SCI. Proper management of these chronic conditions can further reduce medical costs and healthcare burdens. Our findings will lay the foundation for a fully powered randomized controlled trial which would be a reference for the policymakers to develop innovative and comprehensive community healthcare for people with SCI in Hong Kong. As such, this project can meet the service gaps by providing community health support for people with SCI in Hong Kong, who are highly vulnerable (and maybe marginalized) due to their severe life-long disability. It will also echo the Hong Kong Food and Health Bureau's

strategic direction to stepping up the control of modifiable risk factors (e.g., physical inactivity) for non-communicable diseases, as well as to support the Hong Kong Government's commitment to "fighting against non-communicable diseases on all fronts and alleviating its burden" [21].

b) Aims and Hypotheses to be Tested

This study **aims** to evaluate the feasibility, acceptability, and preliminary effects of an eight-week Physical-Psychological Integrative (PPI) online-group intervention on physical inactivity, depression, and chronic pain for people with SCI living in the community.

Research hypotheses: when compared with those the control group (online telephone didactic education), participants in the PPI intervention group will indicate significantly greater:

1. Improvements in *the minutes of performing the moderate-to-rigorous physical activity* as well as the *number of days they are physically active* for a total of at least 60 minutes or more per day at post-intervention, and 3-month follow-up;
2. Improvements *in depression and chronic pain* at post-intervention, and 3-month follow-up;
3. Improvements in *mindfulness skills and quality of life* at post-intervention, and 3-month follow-up.

c) Plan of investigation:

(i) Study method/design

An open-label, two-arm pilot RCT will be conducted to investigate the effects between the intervention (PPI online-group intervention) and the control group (brief online didactic education) for people with SCI living in the community over a 3-month follow-up. Qualitative interviews with focus groups involving participants in the PPI intervention group will be conducted post-intervention,

to explore participants' views about acceptance, strengths and limitations, and suggested improvements of the intervention. Participants' recruitment and other study procedures are summarized in a flow chart (Appendix 1) according to the CONSORT statement.

(ii) Participants (subjects)

Study participants will be recruited from the Hong Kong Direction Association for the Handicapped and other community centers in Hong Kong, where groups of people with physical impairments can be reached. The Hong Kong Direction Association for the Handicapped (https://www.4limb.org/) is a **typical non-government organization that serves a large group of people with severe physical impairment/handicaps in Hong Kong.** According to personal communication with the President of the Association, there are approximately a thousand members of the association and over 30% of them are SCI survivors. Emails/phone call invitations will be sent to potential participants and advertising posters will also be used to encourage them to participate.

Eligibility:

People with SCI will be *included* if they are: (1) at least 18 years old; (2) currently living in the community and having SCI for more than 6 months; (3) complete injury at the C6 or below or incomplete injury at any level; (4) having a computer/smartphone with audio-speaking function and Zoom software, and internet access in a secure place; (5) using a wheelchair for at least 2 hours a day and having approval from their physicians to perform exercises; (6) having no problems in hearing, verbal communication and vision; and (7) able to communicate in Cantonese and to provide informed consent.

People with SCI will be *excluded* if they are: (1) presenting with any significant cognitive impairment or brain injury; (2) engaged in ongoing psychotherapy or

any other physiotherapy/exercise/relaxation interventions; (3) physically active for more than 150 minutes per week; and (4) experiencing significant psychotic symptoms, substance misuse or medically unfit for the exercise and psychological programme as diagnosed by their physicians.

Sample size

As this proposed study is to develop and evaluate the feasibility, acceptability, and preliminary effectiveness of the PPI intervention, a widely acceptable sample size (i.e., 30 participants in each study group) for a preliminary analysis to examine intervention feasibility and to estimate a between-group effect will be adopted [22]. Considering a 20% attrition rate, 36 people with SCI per study group (i.e., 72 people in total) will be recruited. A purposive sampling by key informants (containing both participants who completed the whole intervention sessions and those who withdrew from the intervention period) will be conducted for focus-group interviews (i.e., 4-5 participants per group), until saturation of the information was reached.

Randomization and masking

After the completion of the baseline assessment, eligible participants will be randomly allocated to two study groups. The random allocation will be conducted by an independent researcher with an external randomization service (i.e., sealedenvelope.com), using random permuted block sizes (i.e., 4 or 6) to ensure balanced group numbers. Participants will not be blinded due to the nature of the intervention. Participants will be reminded to keep the intervention content confidential and not discuss this with participants in the control group to avoid contamination. Assessor-blind is not adopted as measurements are self-reported. Assistance provided by caregivers and research assistants might be needed for those who have difficulties in filling out surveys.

(iii) Interventions

The Intervention Group--The eight-week Physical-Psychological Integrative online-group intervention

Part I: Physical exercise. A guiding video of performing exercises was specifically developed for the SCI population by two experienced physiotherapists in Hong Kong. The video mainly includes warm-up, mobilization, strength exercises, aerobic exercises, and cool-down sessions.

Part II: An eight-week online intervention. The PPI intervention includes eight weekly online groups (with 4-5 participants in a group) sessions (with each session lasting for 60-90 minutes). At the beginning of each online group meeting, the intervention provider will use motivational interviewing techniques [23] to promote participants' adherence to the physical activity program mentioned in Part I, followed by online group psychological intervention. The content of the intervention will be adapted from the mindfulness-based stress reduction proposed by Kabat Zinn (1992), practice guideline of 'mindfulness for health' to relieve pain, reduce stress, and restore well-being [24]. The content of the intervention includes eight themes/sessions (the details of the intervention content are shown in Appendix 2): Session 1- Orientation and engagement; Session 2- Awareness and Acceptance; Session 3- Non-judgement; Session 4- Stay present and let go; Session 5- Our thoughts are not real & Response without reacting; Session 6- Empowerment of self-management and discuss pain management; Session 7- Seek out pleasant things and social support; Session 8- Review the intervention and end the programme. Two audio-tape recorded sessions (i.e., 20 minutes each) of mindfulness practice will be given to the participants for their daily practice. The intervention will be carried out by an experienced social worker trained in psychology, motivational interviewing, and mindfulness-based stress reduction.

The control group

Participants in the control group will receive a video call (approximately 60 minutes each week for eight weeks) from the trained research assistant with psychological background, i.e., to provide general physical and psychological suggestions (e.g., encouragement of performing physical activities, and communication skills with family members/friends, and engagement in the community life). This is to control the contact effects of the PPI intervention.

Study attrition and data fidelity

The research assistant will contact the participants via phone/WhatsApp and encourage them to attend the online meetings as scheduled. Fitbit wristband activity trackers will be used as incentives for the participants to remain in the study. The group intervention attendance and dropout of participants will be monitored by the intervention facilitator, and a logbook will be used to record their attendance. Intervention sessions will be audio-taped with participants' consent for reviews and discussion of improvements among the research team. A researcher independent of the study execution will be responsible for periodic monitoring of data collection progress, quality, and safety. All adverse events will be identified, reported, and handled cautiously.

Ethical considerations

Ethical approval will be obtained from the Hong Kong Polytechnic University. This study will be performed in accordance with the Helsinki Declaration. Written informed consent will be obtained from all participants before the baseline assessments of the study, and will also be obtained from purposely sampled participants prior to all focus-group interviews. Participation is entirely voluntary for people with SCI and they can withdraw from the research at any time without any penalty. Participants' identities and data (information

collected and videotape records) will be protected by maintaining anonymity and confidentiality. Only authorized personnel can access the data for analysis.

(iv) Data processing and analysis

Feasibility and acceptability

The feasibility assessment of subject recruitment and follow-up will include (1) time taken to recruit the target sample size; (2) recruitment rate: the number of eligible participants approached who agree to consent divided by all subjects eligible to participate; (3) retention rate: the number of participants who complete the study divided by all participants who agree to consent; (4) drop-out rate: the number of participants who dropped out after randomization divided by all participants who agree to consent; and (5) reasons for dropping out will be collected from participants who dropped out.

The acceptability of the intervention will be indicated by (1) adherence rate: the number of PPI intervention sessions practiced divided by the total number of required sessions; (2) adverse events record associated with PPI intervention; and (3) satisfaction: participants' satisfaction with the intervention will be assessed with the Client Satisfaction Questionnaire [25]. The acceptability of the intervention will also be assessed by focus-group interviews with a purposive sample of participants who completed the whole intervention sessions and those who withdrew from the intervention period. Participants' perspectives about the acceptability, strengths, and limitations of the study and suggestions on the improvements of the interventions will be explored.

Preliminary effectiveness

All participants will be assessed at pre-, post-intervention, and 3-month follow-up by the research assistant. A self-designed form will be used to collect data on participants' socio-demographics and medical history, including age, gender,

employment, marital status, ethnicity, cause, level of injury, and time since the injury. The following outcomes/instruments will be used to assess the preliminary effects of the PPI intervention. Effect sizes (e.g., Cohen's d) and confidential intervals will be calculated to estimate the sample size for a future fully powered randomized controlled trial.

Primary outcomes include

Leisure-time Physical Activity. Participants' leisure-time moderated-to-rigorous physical activity will be assessed by using the Fitbit Orb (San Francisco, IL) which is a wearable, display, triaxial accelerometer that pairs with Bluetooth with compatible smartphones. It can monitor the heartbeat rate of users for each minute. Participants will be asked to wear the Fitbit tracker for more than 10 hours a day (defined as daily adherence) throughout the study period. This accelerometer-based wristband activity tracker has shown its validity in measuring physical activity in cohorts with SCI [26].

Depression. The 9-item Patient Health Questionnaire will be used to measure the participants' depression level. Participants will be asked to rate "how often they were bothered by specific symptoms over the last two weeks" with each item rating from 0 (not at all) to 3 (nearly every day). Higher scores indicate greater symptoms of depression. The scale has indicated satisfactory reliability in the SCI population [16].

Chronic Pain. An 11-point numerical pain rating scale will be used to access pain intensity (in the past week), where zero means no pain and 10 means the worst imaginable pain. The scale has demonstrated good reliability in the Chinese SCI population [20].

Secondary outcomes include:

Exercise Efficacy. The 10-item Chinese version of the self-efficacy for exercise will be used to evaluate participants' confidence level (from 0 not confident to 10 very confident) regarding engaging in regular exercise. The average scores of ten items will be calculated, with the higher scores indicating greater exercise efficacy. The scale demonstrated good reliability and validity in Chinese people using wheelchairs [27].

Mindfulness. The 39-item Five Facet Mindfulness Questionnaire will be used to measure the five aspects of mindfulness including observing, describing, acting with awareness, non-judgmental and non-reactive. Answering on a five-point Likert scale (from 1 never to 5 always true), higher scores denote greater levels of mindfulness. Satisfactory reliability was found for each subscale in the SCI population [28].

Quality of life. The 26-item World Health Organization Quality of Life Brief Scale will be used for assessing participants' quality of life in four domains including physical health, psychological health, social relationships, and environment. Higher scores indicate greater perceived quality of life with grading on a five-point Likert scale. This scale has demonstrated strong psychometric characteristics of good reliability in the SCI population [28].

Data collection and processing

With participants' written consent, pre-intervention test will be conducted face-to-face (or online if participants cannot come to the study site) by filling out the questionnaires by the participants by participants on their own. Assistance for those who have difficulties in filling out surveys will be provided by their caregivers or research assistants. At post-intervention and 3-month follow-up, the research assistant will use the same set of outcome questionnaires but the evaluation will be online. Focus groups will be conducted online as described above in the 'feasibility and acceptability' section. With their consent, the interviews (and intervention sessions) will be audio-taped for content analysis.

Data analysis

All quantitative data from the pre- and post-tests will be numerically coded, summarized (with descriptive statistics), and analyzed using IBM SPSS 26. Both intention-to-treat and per-protocol analyses will be performed. Effect sizes of between-group comparisons will be estimated using Cohen's d which is applicable for small sample sizes [29]. Comparison of baseline information between two study groups will be performed by t-test, Mann-Whitney U, and/or Chi-square tests depending on the nature of the data. The generalized estimating equation will be used to assess the changes in the outcome measurements between groups over time, followed by contrast tests. Co-variance (if any found in the baseline comparison) will be adjusted during the analysis of intervention effects. Subgroup analysis will be conducted for certain factors (e.g., complete and incomplete injury or intervention completion and non-completion). Level of significance is set at $p<0.05$ (2-tailed).

Focus group interview data will be content analyzed. Tape-recorded interviews will be transcribed into Cantonese by one research assistant and cross-checked by the other research assistant. Codes will be discussed and agreed between our team members, combined to form categories/subcategories, and explained with verbatim data. Any discrepancy in coding/category will be discussed and final themes/subthemes will be confirmed by our team.

(d) Key references

1. Alizadeh A, Dyck SM, Karimi-Abdolrezaee S: Traumatic Spinal Cord Injury: An Overview of Pathophysiology, Models and Acute Injury Mechanisms. *Front Neurol* 2019, 10:282-282.
2. March ICfCoSCIPJI: Global summary of spinal cord injury, incidence and economic impact. 2004.
3. Margolis JM, Juneau P, Sadosky A, Cappelleri JC, Bryce TN, Nieshoff ECJAopm, rehabilitation: Health care resource utilization and medical costs of spinal cord injury with neuropathic pain in a commercially insured population in the United States. 2014, 95(12):2279-2287.

4. Hu Y, Mak JN, Wong YW, Leong JC, Luk KDJJorm: Quality of life of traumatic spinal cord injured patients in Hong Kong. 2008, 40(2):126-131.
5. Ginis KM, Jetha A, Mack D, Hetz SJSC: Physical activity and subjective well-being among people with spinal cord injury: a meta-analysis. 2010, 48(1):65-72.
6. Quel de Oliveira C, Refshauge K, Middleton J, de Jong L, Davis GMJJoN: Effects of activity-based therapy interventions on mobility, independence, and quality of life for people with spinal cord injuries: a systematic review and meta-analysis. 2017, 34(9):1726-1743.
7. Khazaeipour Z, Taheri-Otaghsara S-M, Naghdi MJTiscir: Depression following spinal cord injury: its relationship to demographic and socioeconomic indicators. 2015, 21(2):149-155.
8. Bombardier CH, Richards JS, Krause JS, Tulsky D, Tate DGJAopm, rehabilitation: Symptoms of major depression in people with spinal cord injury: implications for screening. 2004, 85(11):1749-1756.
9. Hadjipavlou G, Cortese AM, Ramaswamy B: Spinal cord injury and chronic pain. *BJA Education* 2016, 16(8):264-268.
10. Avluk OC, Gurcay E, Gurcay AG, Karaahmet OZ, Tamkan U, Cakci AJAoSm: Effects of chronic pain on function, depression, and sleep among patients with traumatic spinal cord injury. 2014, 34(3):211-216.
11. Best KL, Routhier F, Sweet SN, Lacroix E, Arbour-Nicitopoulos KP, Borisoff JF: Smartphone-Delivered Peer Physical Activity Counseling Program for Individuals With Spinal Cord Injury: Protocol for Development and Pilot Evaluation. *JMIR Res Protoc* 2019, 8(3):e10798.
12. Bombardier CH, Dyer JR, Burns P, Crane DA, Takahashi MM, Barber J, Nash MS: A tele-health intervention to increase physical fitness in people with spinal cord injury and cardiometabolic disease or risk factors: a pilot randomized controlled trial. *Spinal Cord* 2020.
13. Boldt I, Eriks-Hoogland I, Brinkhof MWG, de Bie R, Joggi D, von Elm E: Non-pharmacological interventions for chronic pain in people with spinal cord injury. *Cochrane Database of Systematic Reviews* 2014(11).
14. Fann JR, Crane DA, Graves DE, Kalpakjian CZ, Tate DG, Bombardier CH: Depression Treatment Preferences After Acute Traumatic Spinal Cord Injury. *Archives of Physical Medicine and Rehabilitation* 2013, 94(12):2389-2395.
15. Heutink M, Post MW, Luthart P, Schuitemaker M, Slangen S, Sweers J, Vlemmix L, Lindeman EJJorm: Long-term outcomes of a multidisciplinary cognitive behavioural programme for coping with chronic neuropathic spinal cord injury pain. 2014, 46(6):540-545.
16. Hearn JH, Finlay KAJSC: Internet-delivered mindfulness for people with depression and chronic pain following spinal cord injury: a randomized, controlled feasibility trial. 2018, 56(8):750-761.

17. Hearn JH, Cross A: Mindfulness for pain, depression, anxiety, and quality of life in people with spinal cord injury: a systematic review. *BMC Neurology* 2020, 20(1):32.
18. Vall J, Costa CMdC, Pereira LF, Friesen TTJAdn-p: Application of International Classification of Functioning, Disability and Health (ICF) in individuals with spinal cord injury. 2011, 69(3):513-518.
19. Li Y, Chien WT, Bressington D: Effects of a coping-oriented supportive programme for people with spinal cord injury during inpatient rehabilitation: a quasi-experimental study. *Spinal Cord* 2020, 58(1):58-69.
20. Li Y, Bressington D, Chien W-TJD, rehabilitation: Pilot evaluation of a coping-oriented supportive program for people with spinal cord injury during inpatient rehabilitation. 2019, 41(2):182-190.
21. Strategy and Action Plan to Prevent and Control Non-communicable Diseases in Hong Kong. Food and Health Burean, Hong Kong.
22. Whitehead AL, Julious SA, Cooper CL, Campbell MJ: Estimating the sample size for a pilot randomised trial to minimise the overall trial sample size for the external pilot and main trial for a continuous outcome variable. 2016, 25(3):1057-1073.
23. Richardson LJJoCN: Motivational interviewing: helping patients move toward change. 2012, 29(1):18-24.
24. Burch V, Penman D: Mindfulness for health: a practical guide to relieving pain, reducing stress and restoring wellbeing: Hachette UK; 2013.
25. Myaskovsky L, Gao S, Hausmann LRM, Bornemann KR, Burkitt KH, Switzer GE, Fine MJ, Phillips SL, Gater D, Spungen AM *et al*: How Are Race, Cultural, and Psychosocial Factors Associated With Outcomes in Veterans With Spinal Cord Injury? *Arch Phys Med Rehabil* 2017, 98(9):1812-1820.e1813.
26. Veerubhotla A, Hong E, Knezevic S, Spungen A, Ding D: Estimation of Physical Activity Intensity in Spinal Cord Injury Using a Wrist-Worn ActiGraph Monitor. *Archives of Physical Medicine and Rehabilitation* 2020, 101(9):1563-1569.
27. Hsu CY, Moyle W, Cooke M, Jones C: Seated T'ai Chi in Older Taiwanese People Using Wheelchairs: A Randomized Controlled Trial Investigating Mood States and Self-Efficacy. *J Altern Complement Med* 2016, 22(12):990-996.
28. Hearn JH, Finlay KA: Internet-delivered mindfulness for people with depression and chronic pain following spinal cord injury: a randomized, controlled feasibility trial. *Spinal Cord* 2018, 56(8):750-761.
29. Bowring A, Telschow FJE, Schwartzman A, Nichols TE: Confidence Sets for Cohen's d effect size images. *Neuroimage* 2021, 226:117477.

Appendix 1. Flow chart of the study.

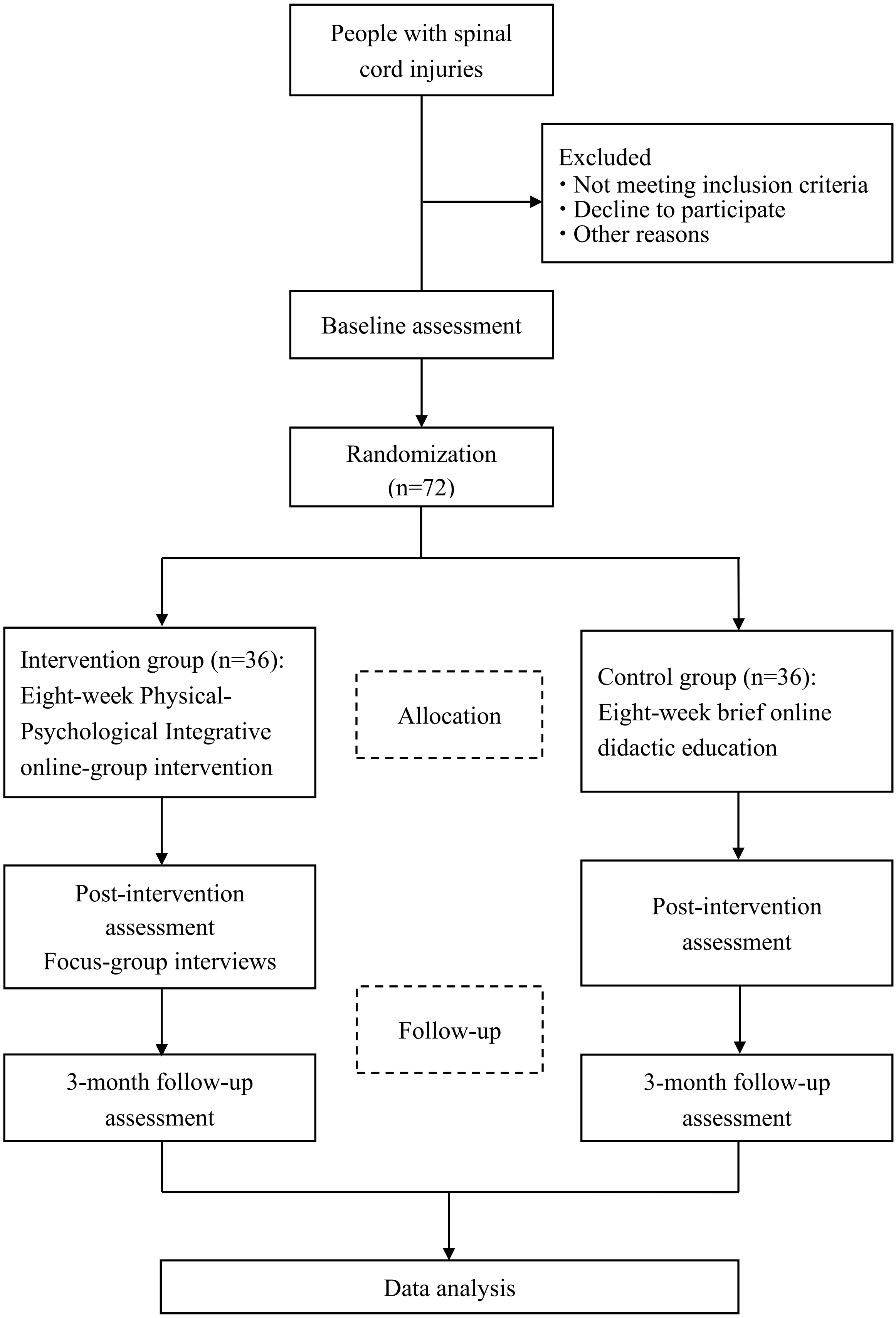

Appendix 2. Details of the intervention.

Session	The theme of the session	Objectives	Content
**1st session**	Orientation and engagement	- Get familiar with the use of the online platform<br>- Understand the aim/objectives of the intervention<br>- Give practical information about group meetings<br>- Encourage participants' attendance at the programme<br>- Review and discuss the physical activity practice	- Introduction of the online platform, facilitate and group members.<br>- Explain the aim of the intervention to increase their activity levels, and reduce depression and chronic pain<br>- To state the group rules and meeting details (attendance, respecting others and being quiet when practising)<br>- Homework: physical activity practice
**2nd session**	Awareness and Acceptance	- Discuss the practice of physical activities<br>- Practice body scans draw notice to the body sensations, rather than attending to thoughts, ideas or fears about the sensations<br>- Discuss disease management in the community	- Body scans of guiding the participants to draw their attention to various areas of the body; move awareness systematically through each area of the body, and notice sensations of the body in a precise and detailed manner, as opposed to attending to thoughts, ideas or fears about this sensation.<br>- To discuss disease management in the community, and the importance of psychosocial care<br>- Homework: physical activity, body scan, and mindful breathing meditation
**3rd session**	Non-judgement	- Discuss the practice of physical activities<br>- Explore present moment experience<br>- Focused attention on breathing with acceptance and a non-judgmental attitude<br>- Practice breath awareness meditation	- Breath awareness meditations began with a broad awareness of the bodily experience of breathing, becoming increasingly focused on more subtle aspects of breathing and encouraged participants to notice when their attention wandered away from the meditation<br>- Homework physical activity, body scan and mindful breathing
**4th session**	Stay present and let go	- Discuss the practice of physical activities<br>- Practice the attitude: Stay present and let go<br>- Increase acceptance and compliance with medication<br>- Practice mindfulness movements and	- Mindfulness movement and mindful sitting (with breath awareness)<br>- Observe experience with stay present attitude and observe body sensations, feelings and thoughts<br>- Homework: mindful movement and mindful sitting, physical activity

		mindful sitting	
**5th session**	Our thoughts are not real & Response without reacting	- Discuss the practice of physical activities<br>- Aware of the thoughts—alternative perspectives of seeing your thoughts and sensations<br>- Integrate mindfulness in managing stress and daily difficulties<br>- Recognize recurring thoughts and stand back from them, without questioning them<br>- Discuss stigma and how to react to discrimination	- Continue the practice of body scans and mindful breathing<br>- Recognize negative thoughts<br>- An alternative response to negative thoughts is "mindful, and response without reacting"<br>- Discuss stigma and how to react to negative thoughts and behaviours to discrimination<br>- Homework: recognize negative thoughts and practice mindfulness response without reacting
**6th session**	Empowerment of self-control	- Discuss the practice of physical activities<br>- Use a mindfulness attitude for problem-solving and communication with mindfulness attitude	- Discuss participants' problems, and how to perform problem-solving<br>- Learn to use a mindfulness attitude for communication<br>- Homework: practice problem-solving skills and mindfulness communication
**7th session**	Seek out pleasant things	- Encourage the exploration of pleasant things in life<br>- Practice kind mediation	- Seek out the pleasant things in life that pain and other sufferings may have prevented from appreciating, aa and find a particular time in a day to find/do things positively.<br>- Meditation that encourages kindness to themselves and others and relaxes into the pain instead of being distressed by it
**8th session**	Review the programme and end the session	- To encourage participants to persist in practising leisure time activities, and to practice, skills learnt in the mind-body intervention<br>- To build social networks and share the experience with peers<br>- To end the programme	- Invitation to outcome assessment<br>- Importance of social networking and sharing experience with peers<br>- Review the mindfulness skills and encourage the continuous practice of physical activity and mind-body interventions<br>- To end the session
